# Sex-Dimorphic Association of Plasma Fatty Acids with Cardiovascular Fitness in Young and Middle-Aged General Adults: Subsamples from NHANES 2003–2004

**DOI:** 10.3390/nu10101558

**Published:** 2018-10-20

**Authors:** Pei-Ling Tsou, Chang-Jiun Wu

**Affiliations:** Department of Genomic Medicine, University of Texas, MD Anderson Cancer Center, 1901 East Road, 3SCR5.4101, Houston, TX 77054, USA; peiling.tsou@gmail.com

**Keywords:** fatty acids, omega-6 polyunsaturated fatty acid, very long chain saturated fatty acid, cardiovascular disease, cardiovascular fitness, plasma

## Abstract

To explore the potential association of plasma fatty acids (FAs) and cardiovascular fitness level (CVFL), data of 449 subjects from 2003–2004 National Health and Nutrition Examination Survey (NHANES) were analyzed. Among these 249 men and 200 women, aged 20–50 years (33.4 ± 8.4 year, mean ± Standard Deviation), 79 low, 166 moderate and 204 high CVFL were categorized by age- and gender- specific percentile, respectively. Twenty-four fatty acids were quantified from fasting plasma. Higher levels of 2 very long-chain saturated FAs (VLSFAs): Arachidic acid (AR1, C20:0) and Docosanoic acid (DA1, C22:0) as well as 2 *n*-6 polyunsaturated FAs (PUFAs): Arachidonic acid (AA, C20:4*n*-6) and Docosatetraenoic acid (DTA, C22:4*n*-6) were observed in the subjects with low CVFL. Notably this association exists only in men. Estimated maximal oxygen consumption (VO_2max_), the marker for cardiorespiratory fitness, was used for further regression analysis. After the adjustment of potential confounding factors (age, smoking, hypertension status, body mass index (BMI), insulin resistance status, and C-reactive protein (CRP), AA was the only FA correlated with low VO_2max_ in women; while in men AR1, DA1, AA, and DTA remain negatively associated with VO_2max_. This preliminary analysis suggests a sex-dimorphic relationship between these plasma VLSFAs and *n*-6 PUFAs with CVFL and merits further investigation.

## 1. Introduction

Cardiovascular fitness, the ability to carry out moderate to high intensity exercise over a prolonged period of time, is measured as maximal oxygen consumption (VO_2max_) [1]. Numerous epidemiological studies have indicated an inverse association between cardiovascular fitness and coronary heart disease (CHD) or all-cause mortality in healthy participants [2,3,4,5]. The benefits of higher midlife cardiovascular fitness are reported to extend well into the later part of life, up to 4 decades [6].

Plasma FAs serve as physiologically important energy substrates and their release from the adipose tissue by lipolysis is regulated according to the energy demands of the body. In fact, under physiologic conditions, plasma free FAs provide 60–70% of the adenosine triphosphate required by myocardial metabolism [7]. Moreover, FAs are active components of biological membranes. An elevated level of free fatty acids (FFAs) with subsequent increased β-oxidation in the cardiomyocytes can be detrimental for the heart in numerous ways. For example, increased FFAs can lead to damage to the plasma membrane and disturbances of the ion channels of the cardiomyocytes [7].

While fatty acids are known to play important roles in cardiovascular pathophysiology [8,9,10], limited data are available on the relation between plasma fatty acids and cardiovascular fitness. A large body of literature studying FAs effects on cardiovascular health relies on dietary assessment from food frequency questionnaire instead of direct measurement of FAs from plasma. Dietary assessment of individual fatty acid intake, although been used mostly, do not reflect the inter-individual differences in absorption, de novo synthesis, metabolism and other genetic variations leading to different levels of circulating FAs. Measurement of plasma as an aggregate of both dietary and biological processed of FAs may be more appropriate for assessing FAs effects as biomarkers [11].

Despite increasing appreciation of the values of circulating FAs for their biomedical relevance, many studies measure only the total amount but not individual FA levels. However, since different fatty acids have distinct biochemical properties and can execute various biological and metabolic tasks, it is possible that different FAs have their specific changes under diverse pathophysiological settings. For example, γ-linolenic acid (GLA) and dihomo-γ-linolenic (DGLA) have been shown to be positively associated, whereas levels of linoleic acid (LA), eicosapentaenoic acid (EPA) and docosahexaenoic acid (DHA) are inversely associated, with biomarkers of inflammation [12,13]. Additionally, levels of palmitoleic acid are reported to be positively associated, while levels of LA are inversely associated, with ischemic stroke and insulin resistance [14,15]. Likewise, while plasma total FAs concentrations are known to be associated with cardiovascular and metabolic diseases, emerging evidence has begun to demonstrate that levels of certain FAs may be more informative regarding cardiovascular situation than total amount of FAs. For instance, low levels of C20:4*n*-3 (eicosatetraenoic acid) and in particular high levels of C18:1*n*-7 (vaccenic acid) were significantly associated with total mortality in heart failure population [16]. Specifically, among *n*-6 PUFAs levels in heart failure patients, there was a decrease in the content of C18:2*n*-6 (linoleic acid), increased C22:5*n*-6 (DTA) and C22:4*n*-6 (DPA *n*-6) and no changes in C20:4*n*-6 (AA) [16].

Given the importance of cardio-metabolic functions exerted by plasma FAs, it is possible that some circulating FAs could potentially serve as biomarkers reflecting cardiovascular health status. The primary goal of this study was to examine the potential associations of plasma FAs level with CVFL, a modifiable risk factor that has been linked to long-term morbidity and mortality.

## 2. Materials and Methods

### 2.1. Study Population

National Health and Nutrition Examination Survey (NHANES) is a cross-sectional observational study conducted by the National Center for Health Statistics of the Centers for Disease Control and Prevention that uses a stratified, multistage probability design to obtain a nationally representative sample of the U.S. population [17]. In the NHANES 2003–2004 cohort (*n* = 10,122), 1845 people (age >20 years old) have measurement of plasma concentrations of fatty acids, and 2809 people completed a submaximal treadmill exercise test. Current analyses include men and women who have valid data for both examinations (*n* = 449).

### 2.2. Examinations and Laboratory Measurements

#### 2.2.1. Cardiorespiratory Fitness Assessment

Per NHANES protocol [18], a multiple-stage submaximal treadmill exercise test was employed to estimate maximal oxygen consumption (VO_2max_), the marker for cardiorespiratory fitness. Briefly, the exercise test included a two-minute warm-up on the treadmill, two separate three-minute submaximal exercise stages and a two-minute cool-down. Heart rate associated with known workloads at the end of each stage was used to estimate VO_2max_. Inclusion criteria for exercise testing were based on medical conditions, medication and physical limitations determined during household interviews, and limited to adults 18–49 years old who did not have any known cardiac conditions. Based on gender and age specific criteria, the estimated VO_2max_ is also categorized and reported as “low”, “moderate” or “high” level of cardiovascular fitness in the present dataset. The reference cut-points used for adults are based on data from the Aerobics Center Longitudinal Study (ACLS) [19]. Low level of coefficients of variance (CV) fitness is defined as an estimated VO_2max_ below the 20th percentile of the ACLS data of the same gender and age group; moderate fitness is defined as a value between the 20th and 59th percentile, and high fitness level is defined as at or above the 60th percentile.

#### 2.2.2. Measurement of Plasma Fatty Acids

Fasting plasma fatty acid concentrations were measured by gas chromatography-mass spectrometry (GC-MS) based on a modification of the method of Lagerstedt et al. [20]. All unsaturated fatty acids are assumed to be in the cis-configuration.

#### 2.2.3. Anthropometric Measures

A wall-mounted stadiometer and a digital floor scale were used to measure height and weight, and calibrated as previously described [21].

#### 2.2.4. Smoking Status

Serum cotinine, a major metabolite of nicotine and a biomarker of tobacco smoke exposure, was measured by an isotope dilution-HPLC (high performance liquid chromatography)/atmospheric pressure chemical ionization tandem mass spectrometry [22]. Individuals with serum cotinine levels >10 ng/mL were coded as being current smokers.

#### 2.2.5. Blood Pressure and Hypertension Status

After resting quietly in a sitting position for 5 min and determining the maximum inflation level, three and sometimes 4 blood pressure (BP) determinations (systolic and diastolic) were taken. The arithmetic mean of 3 or 4 measurements were calculated as mean systolic and diastolic pressure. Hypertension was defined if the mean systolic ≥130 mmHg or the mean diastolic ≥80 mmHg.

#### 2.2.6. Assessment of Lipid Profile, Insulin Resistance Status and C-Reactive Protein

Detailed instructions on specimen collection and processing can be found on the NHANES website [17]. Briefly, blood samples were obtained after a 6- or 9-hour fasting by trained medical personnel in mobile examination centers. Total cholesterol is measured enzymatically in serum or plasma in a series of coupled reactions that hydrolyze cholesteryl esters and oxidize the 3-OH group of cholesterol. HDL (high-density lipoprotein) is measured using direct immunoassay. Triglycerides are measured enzymatically in serum using a series of coupled reactions. LDL (low-density lipoprotein)-cholesterol is calculated from measured values of total cholesterol, triglycerides, and HDL-cholesterol according to the Friedewald calculation:

[LDL−cholesterol]=[total cholesterol]−[HDL−cholesterol]−[triglycerides/5]

where [triglycerides/5] is an estimate of VLDL (very low-density lipoprotein)-cholesterol and all values are expressed in mg/dL. Plasma glucose concentration was determined by hexokinase enzyme method. Plasma insulin concentrations were measured with Tosoh AIA-PACK IRI (Toyama, Japan) two-site immunoenzymometric assay. C-peptide concentrations were measured using 125I-labeled radioimmunoassay. HOMA-IR (homeostatic model assessment for insulin resistance the product of fasting plasma glucose and insulin concentrations divided by 22.5 [mM×μIU/mL/22.5]) was used as a composite index for insulin resistance status. C-reactive Protein (CRP) is quantified by latex-enhanced nephelometry.

### 2.3. Statistical Methods

Descriptive statistics including means, standard deviations, and range for continuous variables and frequencies for categorical variables were reported. To test if the levels of an individual demographic variable are different among three fitness groups, ANOVA (Analysis of Variance) test was used for continuous variables and Fisher’s exact test was used for categorical variables. A two-tailed *p* value < 0.05 was considered statistically significant. The differences of plasma FAs between CVFL groups were assessed by ANOVA followed with False Discovery Rate (FDR) estimation for multiple comparisons correction. An FDR value <0.30 was considered statistically significant. Post-hoc pairwise *t*-test analysis was implemented on significant FAs to understand which groups are different. Univariate and multivariate linear regression models were also implemented with VO_2max_ as dependent variable and each plasma FA as main independent variable. Both VO_2max_ and plasma FA levels were logarithmically transformed due to skewed distribution. In multivariate models, appropriate confounding variables were included. All statistical analyses were conducted using R software (R version 3.3.0, Vienna, Austria).

## 3. Results

### 3.1. Subject Characteristics

Table 1 presents the demographic, anthropometric, and biochemical characteristics of the whole study population, as well as information stratified by 3 CVFL groups—“low”, “moderate”, and “high”—with gender and age specific criteria (see Methods 2.2.1). The mean age of the all subjects was 33.4 years and there were slightly more men (55.5%) than women (44.5%). The mean maximal oxygen uptake (VO_2max_) was 32.5, 38.9, and 48.8 mL/kg/min for men and 26.8, 31.5, and 42.3 mL/kg/min for women categorized as low, moderate, and high CVFL, respectively. The distribution of age and gender among 3 CVFL groups were not significantly different. The percentage of current smoking (defined by serum cotinine level) was similar among 3 CVFL groups. The average waist circumference (WC) and body mass index was 90.6 cm and 25.95 kg/m^2^ while subjects with lower CVFL tended to have higher WC and BMI. Additionally, both average systolic and diastolic blood pressure were significantly higher in the low CVFL group, though the percentage of hypertension (defined by average SBP ≥130 mmHg or average DBP ≥80 mmHg) only showed a trend without statistical significance. Fasting plasma glucose concentration and lipid profile (including total cholesterol, triglyceride, HDL and LDL) did not differ among 3 CVFL groups. Notably, plasma insulin, C-peptide concentrations and derived insulin resistance status (HOMA-IR) were negatively correlated with CVFL. The level of plasma C-reactive Protein (CRP) did not show difference among 3 groups.

### 3.2. Twenty-four Fatty Acid Levels in Fasting Plasma

Plasma concentrations of 24 FAs, including 6 SFAs, 7 MUFAs, and 11 PUFAs from all 449 subjects and levels of 3 CVFL groups and are listed in Table 2. All 24 FAs, including the so-called heart healthy *n*-3 FAs, exhibited negative relationships with CVFL, although only 2 SFAs and 3 *n*-6 PUFAs reached statistical significance. Two VLSFA, Arachidic acid (AR1, C20:0) and Docosanoic acid (DA1, C22:0) as well as one *n*-6 PUFA, Arachidonic acid (AA, C20:4*n*-6), were inversely associated with CVFL (ANOVA FDR < 0.30). None of the derivative variables of interest, including the sum of 24 FAs, the sum of 6 SFAs, the sum of 7 MUFA, the sum of 11 PUFA, the sum of 5 *n*-3 PUFAs, the sum of 6 *n*-6 PUFA, the ratio of *n*-6 to *n*-3 PUFA, or the ratio of C16:1 to C16:0 (Δ9 desaturase index, a surrogate marker of the activity of Stearoyl-coenzyme A desaturase) showed significance difference among the 3 groups.

### 3.3. FAs Levels among 3 CV Fitness Groups Stratified by Sex

**Figure 1:**
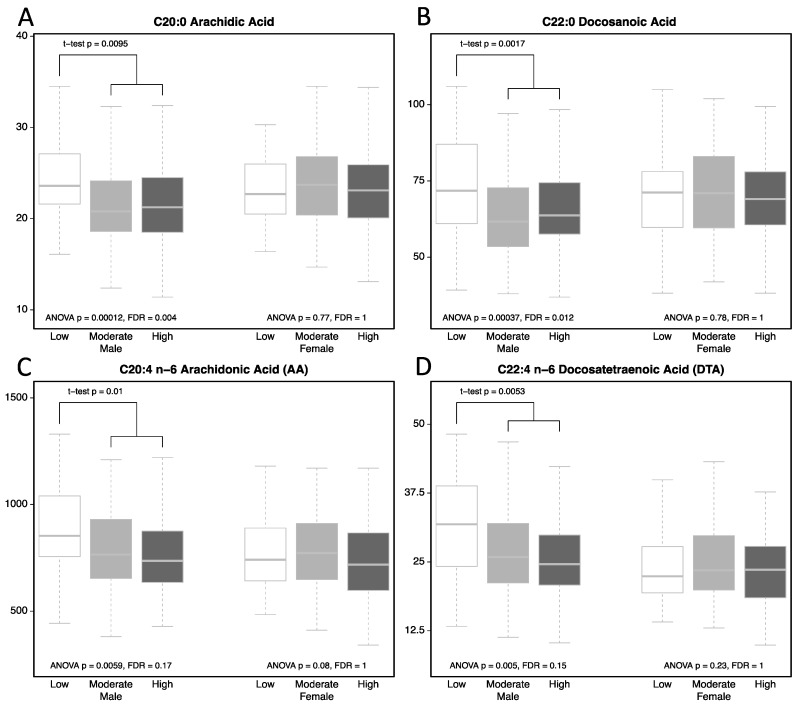
Boxplots of concentrations of 4 FAs (**A**: Arachidic acid, **B**: Docosanoic acid, **C**: Arachidonic Acid, and **D**: Docosatetraenoic Acid) with significant association with CVFL stratified by sex. CVFLs were categorized into low (white), moderate (light gray), and high (dark gray). The levels of Men and Women were shown at the left and right side, respectively. In addition to ANOVA test among 3 CVFL groups, post-hoc pairwise *t*-test analysis was implemented in Men. Significant *t*-test *p* values comparing Low vs. Moderate + High CVFLs in men were shown in the figures. FAs: fatty acids; CVFL: cardiovascular fitness level; ANOVA: Analysis of Variance; FDR: false discovery rate.

Next, we set out to determine whether the relationships of FAs and CVFLs were different in men and women. The levels of 24 FAs among 3 CV fitness groups were further stratified by sex and listed in Table 3. Notably, none of the 24 FAs showed significant difference among 3 CVFL groups in women. On the other hand, the negative associations of CVFL with 2 VLSFAs (AR1, DA1) and one *n*-6 PUFA (AA) were even stronger in the analysis of men then in the whole 449 subjects (Table 3 and Figure 1). Additionally, the negative association of CVFL with D*ocosatetraenoic acid (DTA, C22:*4*n*-6) could only be revealed when examined the levels specifically in men but not from the entire population.

### 3.4. Subjects with Low CVFL Have Higher AR1, DA1, AA, and DTA

Notably, as shown in Figure 1 and Table 4, the levels of Arachidic acid (AR1, C20:0) and Docosanoic acid (DA1, C22:0) were higher only in the low CVFL group, whereas the levels were similar in the moderate and high CVFL groups. As seen in Table 3, for AR1, the mean ± S.D was 26.3 ± 10.4 in the low group, 21.8 ± 4.8 and 21.8 ± 4.7 for the moderate and high CVFLs groups, respectively. For DA1, the mean ± S.D was 76.7 ± 21.1 in the low group, 64.4 ± 16.2 and 65.8 ± 15.6 for the moderate and high CVFLs groups, respectively.

On the other hand, for *n*-6 PUFAs (AA and DTA), there seemed a trend of negative associations across the 3 groups. For AA, the mean ± S.D was 891 ± 255 in the low group, 790 ± 190 in the moderate and 770 ± 203 in the high CVFLs groups. For DTA, the mean ± S.D was 31.6 ± 10 in the low group, 27.3 ± 9.1 in the moderate and 26.2 ± 8.5 in the high CVFLs groups. However, the difference between the moderate and the high CVFL group did not reach statistic significance (*p* = 0.48 for AA, and *p* = 0.35 for DTA, respectively). Thus, higher levels of these 4 FAs were seen only in the low CVFL group.

### 3.5. Univariate and Multivariate Linear Regression Analysis

To further study the relationships of FAs and CVFL, we used VO_2max_ as outcome variable and individual FA levels as independent variable in linear regression models. The 3 CVFLs based on estimated VO_2max_ is gender and age adjusted whereas the estimated VO_2max_ itself is not adjusted. Potential confounding variables were selected taking into account univariate analysis of known factors that reported to be associated CVFLs (data not shown). The effects of various combinations of potential confounding factors were adjusted with multivariate models as described in Table 5. For men, consistently, the negative association of Arachidic acid (AR1), Docosanoic acid (DA1), Arachidonic acid (AA), and Docosatetraenoic acid (DTA) remained significant in all models. For women, on the other hand, Arachidonic acid (AA) was the only FA that showed a negative association with VO_2max_ in multivariate model after adjusting confounding factors.

## 4. Discussion

This study shows for the first-time that plasma concentrations of 2 VLSFAs (AR1, DA1), and 2 *n*-6 PUFAs (AA, DTA) appear to be higher in young and middle-aged general adults with low CVFL, and intriguingly, these associations are sex-dimorphic. The only FA showed negative correlation with VO2max in women is AA and the significance could only be revealed with multivariate analysis after adjusting potential confounders. In contrast, the relationships between VO2max those 4 FAs were seen consistently in men.

The lack of established reference ranges for saturated, trans, monounsaturated and polyunsaturated FA has resulted in the poor interpretability of human research [23]. Most of the researches concerning FAs and cardiovascular health so far have either used frequent food questionnaire to estimate the FAs levels or measured the whole non-esterified fatty acid (NEFA) levels instead of the individual FAs concentrations. Fortunately, there has been an increasing appreciation of the values of measuring circulating FAs to better translate its biomedical relevance [11,24,25,26].

Previous data suggest that plasma levels of FAs and their composition may influence myocardial function. For example, it has been shown that the composition of FAs in chronic heart failure (HF) is important [16]. Specifically, for *n*-6 PUFAs levels in HF patients, there was a decrease in the content of C18:2*n*-6 (linoleic acid), increased C22:5*n*-6 (DTA) and C22:4*n*-6 (DPA *n*-6) but no changes in C20:4*n*-6 (AA). In the current study, these subjects were “apparently healthy” despite of different CVFLs. While we did not find significant difference of *n*-3 PUFAs among CVFL groups, interestingly, we did observe increased DTA in the low CVFL group, as seen in HF patients. AA also negatively correlated with CVFL in our cohort but there was no difference in the more advanced setting (HF). Whether these individual *n*-6 PUFAs have any impact on future cardiovascular events merit further mechanistic study.

The potential beneficial role of *n*-6 PUFAs has been debated [27,28]. Despite the fact that most dietary recommendations agree that SFAs should be at least partially replaced by unsaturated FAs, in particular vegetable PUFAs, the suggestion to increase the intake of PUFAs in general, and *n*-6 PUFAs in particular, continues to be controversial [27,28,29,30]. *n*-6 PUFAs, such as arachidonic acid (AA; 20:4*n*-6), which is the substrate for the synthesis of a variety of proinflammatory and vasoconstrictive molecules, are believed to be proinflammatory [31,32]. On the other hand, despite the concern that *n*-6 fatty acids increase inflammation, evidence from human studies does not support this view [29]. Nevertheless, a recent meta-analysis reviewed 19 randomized controlled intervention trials with 6461 participants and concluded that there was no evidence that increasing omega-6 fat reduces cardiovascular outcomes other than myocardial infarction [33].

Two VLSFAs, Arachidic acid (AR1, C20:0), Docosanoic acid (DA1, or Behenic acid, C22:0) were inversely correlated with CVFL in men in the current study. Several prospective investigations have, however, documented potentially beneficial associations of this unique group, saturated fats with a chain length of 20 or more carbon atoms, with cardiometabolic disease [26,34,35]. VL SFAs are derived mainly from dietary sources and may also be produced endogenously from the elongation of 18:0 to 20:0, 22:0, and 24:0 [36,37]. VLSFAs, however, exhibit distinct functions when compared with other long-chain SFAs. For example, VLSFAs are known to influence liver homeostasis, retinal function, and anti-inflammatory functions [37]. Given the reported beneficial association with various conditions, it is hard to interpret the opposite associations with CVFL. Indeed, in contrast to prospective results, evidence generated from cross-sectional studies has been somewhat mixed [35]. One possible source of the differences between studies could be the methodology of FAs levels assessment from either food questionnaire or the compartment where (such as in serum whole blood, plasma phospholipids or membrane of blood cells) the measurements were done. For example, some studies reported that erythrocyte C24:0 or C26:0 were consistently correlated with atherogenic lipoprotein profiles or diabetes status [38,39]. Further research is needed to elucidate the mechanisms underlying the observed associations.

The sex dimorphism in cardiovascular risks, including lipid metabolism and insulin resistance has been well documented [40,41,42,43,44]. Moreover, it has also been widely accepted that biological sex is an important modifier of the development of cardiovascular disease [45,46]: sex differences are apparent at every level of cardiovascular physiology from action potential duration and mitochondrial energetics to cardiomyocytes and whole heart contractile function [47]. Premenopausal women have reduced levels of CVD compared with their age-matched counterparts [48]. Additionally, baseline FAs levels, FA disposal, and even the clinical effects of FAs supplementation are different in women and man. In general, women have greater nonoxidative FFA disposal and recycling than men [49,50], and the rate of FFA release into plasma is greater in women than men [51]. Additionally, women in the general population have higher circulating DHA concentrations, but lower circulating EPA and DPA concentrations compared with men and this difference is independent of dietary intake [52,53,54]. Interestingly, a recent meta-analysis including 31 eligible randomized controlled trials with a total of 1848 participants reported that a significant improvement in insulin resistance with *n*-3 PUFA supplementation longer than 6 weeks was only seen in women but not in men [55]. Similarly, *n*-3 PUFA supplementation enhances muscle function in older women but not in older men after resistance exercise training [56]. Considering that many known cardiovascular risk factors are sex-dimorphic, and that many aspects of FAs are different in women and men, we thus also further stratified the subjects by gender, and examine the association of CVFL with FA levels separately. Not expectedly, all the associations observed came from men.

Among the 4 FAs of interest, men have higher DTA while the levels other 3 FAs were similar. The absolute concentrations of individual FA *per se* might not be the explanation of the dimorphism. Another possibility is that greater variation of FAs concentration exists in women and masks its biological relevance. This is probably not the case since the coefficients of variance CV (%) of FAs of women were not greater than those of men. Additionally, it has been reported that plasma FAs concentrations were not affected by menstrual cycle [57]. Limited by the nature of the current study, we could not explain the mechanism for the sex-specific associations of FAs and CVFLs. In addition, even though the molecular basis underlying this sex dimorphism remains to be elucidated, further study may provide important insights for gender-specific prevention or intervention strategy.

The present study has a few limitations. FA levels in the current study were measured only in plasma but not other compartments, such as lipoproteins or plasma phospholipids on membranes of blood cells. Therefore, apart from being used as biomarkers, these levels could not provide the whole pictures of FAs metabolism. Also, as a cross-sectional epidemiologic study, these statistical associations do not necessarily imply any causal relationships, and the mechanisms underlying these observations could not be established. Moreover, generalizability of the results seems difficult because the subsample was not chosen randomly but due to the data availability of both CVFLs and FAs. Importantly, although we adjusted for factors that have previously reported to be associated with CVFL [58,59], we cannot exclude the role of residual or unmeasured confounding in the findings. Further researches are required to investigate factors that may modulate effects of these fatty acids.

## 5. Conclusions

In conclusion, in young and middle-aged adults with low CVFL, plasma concentrations of 2 VLSFAs (AR1, DA1) and 2 *n*-6 PUFAs (AA1, DTA) were found to be higher than those of moderate and high CVFL. These associations were seen only in men but not women. Further studies are required to verify the presence of the observed sexual dimorphism in the association of FAs and CVFL, and to investigate the potential impact of these associations on long-term cardiovascular outcome.

## Figures and Tables

**Table 1:** Demographic characteristics of the study population: overall and stratified by cardiovascular fitness levels. CVFLs were categorized into Low, Moderate, and High by estimated VO2max with gender and age specific percentile. Significant values are shown in bold and italic fonts.

	All Subjects		Cardiovascular Fitness Level (CVFL)						
			Low		Moderate		High		ANOVA
	Range (Mean ± S.D)	*n*	Range (Mean ± S.D)	*n*	Range (Mean ± S.D)	*n*	Range (Mean ± S.D)	*n*	*p* value
***VO2max(ml/kg/min)***	20.9–78.9 (39.3 ± 8.7)	448	20.9–37.1 (29.8 ± 4)	79	27.1–43.9 (36 ± 4.4)	166	33.1–78.9 (45.7 ± 7.8)	204	***<0.001***
Age (yr)	20.1–50 (33.4 ± 8.4)	448	20.2–49.9 (31.9 ± 8.3)	78	20.2–49.4 (34.1 ± 7.9)	166	20.1–50 (33.4 ± 8.9)	204	0.161
Gender (M,%)	55.5		53.2	78	60.2	166	52.5	204	¶0.294
Smoking (%)	29.4	449	25.6	78	32.5	166	28.2	202	0.486
***Waist Circumfrence (WC, cm)***	66.3–122 (90.6 ± 11.9)	449	68.4–120 (94.0 ± 12.0)	54	66.5–117 (90.5 ± 11.2)	129	66.3–122 (89.5 ± 12.2)	164	***0.049***
***Body Mass Index (kg/m^2^)***	16.7–40.9 (26.0 ± 4.2)	347	17.6–39.5 (27.3 ± 4.1)	54	16.7–40 (25.9 ± 4.2)	130	17.4–40.9 (25.5 ± 4.2)	165	***0.026***
Hypertension (%)	19.8	349	31.5	54	18.5	130	17.0	165	¶0.060
***Systolic Blood Pressure ( SBP, mmHg)***	87–189 (114 ± 11)	349	98–189 (117 ± 14.7)	54	87–143 (114.7 ± 10.8)	130	89–145 (112.7 ± 10.6)	165	***0.029***
***Diastolic Blood Pressure ( DBP, mmHg)***	40–95 (69 ± 10)	349	51–93 (72.6 ± 10.5)	54	40–95(69.52 ± 9.49)	130	44–95 (68.6 ± 10.1)	165	***0.037***
Plasma Triglyceride (TG, mg/dL )	32–1779 (131 ± 132)	347	32–776 (136 ± 105)	54	35–648 (134. ± 96)	129	33–1779 (128 ± 161)	164	0.892
Plasma HDL (mg/dL)	27–112 (54.6 ± 15.9)	348	29–102 (54.9 ± 17.6)	54	27–103 (52.2 ± 13.4)	130	27–112 (56.5 ± 17)	164	0.065
Plasma LDL (mg/dL)	35–217 (115 ± 33.6)	435	35–201 (118 ± 32.6)	78	41–211 (115 ± 34.6)	160	41–217 (113 ± 33.1)	197	0.466
Plasma Total Cholesterol (mg/dL)	93–426 (193 ± 39.4)	447	93–426 (199 ± 44.1)	79	109–295 (192 ± 39.2)	166	99–306 (192 ± 37.6)	202	0.390
Plasma Glucose (GLU, mg /dL)	63.4–416 (93.5 ± 19.8)	449	72.6–416 (98.3 ± 44.9)	54	75.6–142 (93.7 ± 8.9)	130	63.4–165 (91.9 ± 10.6)	165	0.115
***Plasma C-peptide ( nmol/L )***	0.71–71.1 (9.8 ± 8.8)	446	2.17–42.5 (12.0 ± 8.5)	79	1.16–71.1 (9.9 ± 9)	166	0.71–54.5 (8.9 ± 8.5)	201	***0.023***
***Plasma Insulin( INS, μU/mL )***	0.02–2.77 (0.73 ± 0.35)	446	0.3–2.1 (0.83 ± 0.36)	79	0.24–2.77 (0.74 ± 0.35)	166	0.02–2.47 (0.68 ± 0.34)	201	***0.005***
***HOMA-IR***	0.12–20 (2.36 ± 2.33)	446	0.47–11.3 (2.90 ± 2.24)	79	0.25–20 (2.39 ± 2.31)	166	0.12–16.7 (2.13 ± 2.36)	201	***0.044***
Plasma C-Reactive Protein (CRP, mg/dL)	0.01–16.5 (0.36 ± 0.9)	448	0.02–2.91 (0.44 ± 0.53)	79	0.01–3.41 (0.33 ± 0.42)	166	0.01–16.50 (0.36 ± 1.24)	203	0.670

Abbreviations: VO2max: Estimated maximal oxygen uptake; HOMA-IR: Homeostatic Model Assessment - Insulin Resistance; ANOVA: Analysis of Variance; n: number. ¶ Fisher’s exact test.

**Table 2:** Twenty-four Fatty Acid Levels in Fasting Plasma and 8 derivative variables of interest: overall and stratified by cardiovascular fitness levels

		All Subjects			Cardiovascular Fitness Level (CVFL)						
				Low		Moderate		High		ANOVA	
		Range (Mean ± S.D)	*n*	Range (Mean ± S.D)	*n*	Range (Mean ± S.D)	*n*	Range (Mean ± S.D)	*n*	*p* value	FDR
SFA	C14:0 Myristic Acid	24.8–2410 (137 ± 139)	442	40.2–370 (130 ± 72)	79	24.8–620 (138 ± 93.6)	164	29.2–2410 (140 ± 185)	199	0.88	1.00
	C16:0 Palmitic Acid	1310–8240 (2715 ± 960)	444	1310–8240 (2840 ± 990)	79	1380–7120 (2767 ± 996)	165	1330–7970 (2623 ± 914)	200	0.16	1.00
	C18:0 Stearic Acid	364–1720 (685 ± 180)	444	404–1720 (708 ± 186)	79	364–1470 (694 ± 182)	165	373–1490 (669 ± 175)	200	0.19	1.00
	***C20:0 Arachidic Acid (AR1)***	***6.25–83.7 (23 ± 5.8)***	***433***	***16.1–83.7 (24.9 ± 8.1)***	***78***	***12.4–36.3 (22.6 ± 5)***	***159***	***6.25–43.7 (22.6 ± 5.1)***	***196***	***0.007***	**0.23**
	***C22:0 Docosanoic Acid (DA1)***	***17–171 (68.3 ± 16.5)***	***431***	***38.2–171 (73.5 ± 18.2)***	***78***	***38–129 (66.9 ± 16.4)***	***158***	***17–117 (67.5 ± 15.6)***	***195***	***0.009***	**0.26**
	C24:0 Lignoceric Acid	20.9–132 (54.2 ± 13.3)	430	27.1–112 (56.5 ± 13.7)	76	28.8–132 (53.1 ± 13.2)	161	20.9–104 (54.1 ± 13.2)	193	0.18	1.00
MUFA	C14:1 *n*-5 Myristoleic Acid	0.70–186 (8.8 ± 11.9)	445	0.98–29.4 (7.8 ± 5.3)	79	0.9–75.7 (9.2 ± 10)	165	0.70–186 (8.9 ± 14.8)	201	0.69	1.00
	C16:1 *n*-7 Palmitoleic Acid	39–2500 (238 ± 199)	445	57.7–645 (232 ± 122)	79	51.9–1930 (245 ± 198)	165	39–2500 (236 ± 224)	201	0.86	1.00
	C18:1 *n*-7 cis-Vaccenic Acid	54.1–550 (144 ± 59.1)	435	62.9–550 (148 ± 60.4)	78	59.7–489 (146 ± 57.8)	161	54.1–454 (141 ± 59.7)	196	0.6	1.00
	C18:1 *n*-9 Oleic Acid	885–15800 (2107 ± 1175)	443	885–8560 (2111 ± 926)	79	933–13300 (2157 ± 1193)	164	911–15800 (2065 ± 1249)	200	0.76	1.00
	C20:1 *n*-9 Eicosenoic Acid	2.89–61.1 (13.8 ± 6.7)	443	4.76–61.1 (14.2 ± 7.3)	79	5.09–42 (14 ± 6.8)	164	2.89–46.3 (13.6 ± 6.3)	200	0.72	1.00
	C22:1 *n*-9 Docosenoic Acid	0.21–23.8 (4.5 ± 3.5)	395	0.21–23.8 (4.9 ± 3.7)	65	0.21–18.2 (4.3 ± 3.3)	145	0.21–23.80 (4.39 ± 3.60)	185	0.48	1.00
	C24:1 *n*-9 Nervonic Acid	18.3–162 (76 ± 18.8)	428	38.6–133 (79.3 ± 19.4)	76	34.1–162 (76.7 ± 20.1)	157	18.3–117 (74.1 ± 17.4)	195	0.1	1.00
PUFA	C18:2 *n*-6 Linoleic Acid (LA)	1770–9200 (3503 ± 881)	445	1810–9200 (3571 ± 1000)	79	2130–6970 (3454 ± 844)	165	1770–7450 (3517 ± 863)	201	0.6	1.00
	C18:3 *n*-6 gamma-Linolenic Acid (GLA)	6.25–141 (48.4 ± 23)	442	7.2–136 (48.8 ± 26)	79	10.50–141 (49.2 ± 23.2)	164	6.25–122 (47.5 ± 21.7)	199	0.77	1.00
	C20:2 *n*-6 Eicosadienoic Acid	5.66–123 (22.4 ± 10.3)	443	9.9–52 (22.6 ± 8.5)	79	8.3–64.9 (22.2 ± 9.1)	164	5.66–123 (22.5 ± 11.7)	200	0.92	1.00
	C20:3 *n*-6 Dihomo-gamma-Linolenic Acid (DGLA)	49–716 (156 ± 63.9)	445	53.1–330 (158 ± 51.3)	79	49–612 (158.34 ± 64.57)	165	50.9–716 (153 ± 67.8)	201	0.71	1.00
	***C20:4 n-6 Arachidonic Acid (AA)***	***341–1770 (784 ± 205)***	***445***	***443–1770 (839 ± 232)***	***79***	***380–1390 (796 ± 201)***	***165***	***341–1590 (754 ± 194)***	***201***	***0.005***	**0.16**
	*C22:4 n-6 Docosatetraenoic Acid (DTA)*	*6.72–66.1 (26.2 ± 8.7)*	*445*	*13.3–63.3 (28.2 ± 9.5)*	*79*	*11.3–66.1 (26.8 ± 9)*	*165*	*6.72–58.8 (25 ± 8.1)*	*201*	*0.013*	0.37
	*C22:5 n-6 Docosapentaenoic-6 Acid (DPA n-6)*	*5.31–61.7 (21.5 ± 8.9)*	*445*	*9.5–52.5 (24 ± 9.4)*	*79*	*5.94–56 (21.5 ± 9.1)*	*165*	*5.31–61.7 (20.5 ± 8.3)*	*201*	*0.013*	0.37
	C18:3 *n*-3 alpha-Linolenic Acid (ALA)	16.1–421 (68.2 ± 42.1)	445	23.1–341 (67.1 ± 44)	79	16.1–272 (67.9 ± 37.4)	165	20.2–421 (68.8 ± 45)	201	0.95	1.00
	C20:5 *n*-3 Eicosapentaenoic Acid (EPA)	6.–236 (43.4 ± 28.8)	444	15.7–130 (41.3 ± 23.9)	79	6.60–236 (45.5 ± 33.8)	165	6.03–143 (42.4 ± 26.1)	200	0.46	1.00
	C22:5 *n*-3 Docosapentaenoic-3 Acid (DPA n-3)	12.1–106 (41.5 ± 13.6)	445	17.4–106 (41.4 ± 14.8)	79	15.1–90.4 (42.5 ± 14.2)	165	12.1–98.5 (40.6 ± 12.6)	201	0.42	1.00
	C22:6 *n*-3 Docosahexaenoic Acid (DHA)	43.2–549 (131 ± 62.6)	445	55.1–468 (134 ± 61.3)	79	46.6–549 (135 ± 70.8)	165	43.2–324 (126 ± 55.5)	201	0.35	1.00
	Σ24FFA	13187–78613 (27060 ± 7739)	445	13981–78613 (27800 ± 8222)	79	15832–67736 (27218 ± 7729)	165	13187–67035 (26639 ± 7563)	201	0.5	1.00
	Σ6SFA	1840–10670 (3669 ± 1216)	445	1851–10677 (3829 ± 1246)	79	1977–9243 (3735 ± 1260)	165	1840–10323 (3552 ± 1162)	201	0.16	1.00
	Σ7MUFA	345–17590 (2577 ± 1388)	445	1129–9823 (2592 ± 1078)	79	345–14755 (2631 ± 1421)	165	480–17590 (2527 ± 1470)	201	0.77	1.00
	Σ11PUFA	2375–12529 (4845 ± 1124)	445	2662–12529 (4975 ± 1288)	79	2891–8697 (4818 ± 1075)	165	2375–9739 (4816 ± 1096)	201	0.52	1.00
	Σw3PUFA ¶	92.6–1071 (284 ± 119)	445	142–1030 (284 ± 118)	79	92.6–1071 (291 ± 129)	165	107–786 (278 ± 110)	201	0.57	1.00
	Σw6PUFA §	2255–11499 (4561.6 ± 1048)	445	2472–11499 (4692 ± 1199)	79	2781–8229 (4528 ± 1002)	165	2255–8953 (4539 ± 1024)	201	0.47	1.00
	w6/w3	5.29–37.7 (17.5 ± 4.7)	445	8.2–31.6 (17.7 ± 4.6)	79	5.29–34.4 (17.1 ± 4.7)	165	8.02–37.7 (17.8 ± 4.9)	201	0.41	1.00
	C16:1/C16:0	1.82–37.4 (14.1 ± 5.1)	444	5.99–24.1 (13.8 ± 4.2)	79	3.69–33.8 (13.8 ± 4.9)	165	1.82–37.4 (14.3 ± 5.5)	200	0.62	1.00

All values are in μmol/L. Significant values are shown in bold and italic fonts. Abbreviations: SFA: Saturated Fatty Acid; MUFA: Mono-Unsaturated Fatty Acid; PUFA: Poly-Unsaturated Fatty Acid. ¶ Σw3PUFA: Σ (SSALN_N, SSEPA_N, SSDP3_N, SSDHA_N ). Σ6SFA: Sum of 6 SFAs; Σ7MUFA: Sum of 7 MUFAs; Σ11PUFA: Sum of 11 PUFAs; Σ24FFA: Σ6SFA + Σ7MUFA + Σ11PUFA. ¶ Σw3PUFA: Σ (SSALN_N, SSEPA_N, SSDP3_N, SSDHA_N ); § Σw6PUFA: Σ (SSLNA_N, SSGLA_N, SSED1_N, SSHGL_N, SSARA_N, SSDTA_N, SSDP6_N).

**Table 3:** Twenty-four Fatty Acid Levels in Fasting Plasma and 8 derivative variables of interest among 3 cardiovascular levels further stratified by sex.

		Men								Women							
		Cardiovascular Fitness Level								Cardiovascular Fitness Level							
		Low		Moderate		High		*ANOVA*		Low		Moderate		High		*ANOVA*	
		mean ± S.D	*n*	mean ± S.D	*n*	mean ± S.D	*n*	*p*	*FDR*	mean ± S.D	*n*	mean ± S.D	*n*	mean ± S.D	*n*	*p*	*FDR*
SFA	C14:0 Myristic Acid	141 ± 78.5	42	141 ± 103	99	134 ± 105	103	0.88	1.00	118 ± 62	37	133 ± 77.9	65	145 ± 244	96	0.71	1.00
	C16:0 Palmitic Acid	2999 ± 1098	42	2769 ± 1114	100	2678 ± 971	105	0.25	1.00	2659 ± 828	37	2764 ± 789	65	2561 ± 847	95	0.31	1.00
	C18:0 Stearic Acid	756 ± 212	42	694 ± 203	100	685 ± 185	105	0.14	1.00	654 ± 134	37	694 ± 147	65	640 ± 161	95	0.18	1.00
	***C20:0 Arachidic Acid (AR1)***	26.3 ± 10.4	41	21.8 ± 4.8	99	21.8 ± 4.7	102	***<0.001***	***0.004***	23.2 ± 3.8	37	23.9 ± 5.0	60	23.5 ± 5.4	94	0.77	1.00
	***C22:0 Docosanoic Acid (DA1)***	76.7 ± 21.1	41	64.4 ± 16.2	99	65.8 ± 15.6	101	***<0.001***	***0.01***	70.0 ± 13.8	37	71.0 ± 15.9	59	69.2 ± 15.4	94	0.78	1.00
	*C24:0 Lignoceric Acid*	60.8 ± 14.7	40	53.7 ± 14.2	100	55.9 ± 13.4	102	*0.028*	0.75	51.7 ± 10.8	36	52.0 ± 11.3	61	52.2 ± 12.8	91	0.98	1.00
MUFA	C14:1 n-5 Myristoleic Acid	8.3 ± 5.5	42	9.9 ± 12.0	100	8.4 ± 9.3	105	0.48	1.00	7.3 ± 5.1	37	8.1 ± 5.8	65	9.4 ± 19.1	96	0.69	1.00
	C16:1 n-7 Palmitoleic Acid	244 ± 125	42	255 ± 237	100	246 ± 271	105	0.95	1.00	219 ± 118	37	229 ± 112	65	226 ± 169	96	0.93	1.00
	C18:1 n-7 cis-Vaccenic Acid	160 ± 75.5	41	147 ± 64.1	99	143 ± 62.4	103	0.37	1.00	135 ± 33.4	37	145 ± 46.4	62	139 ± 56.8	93	0.58	1.00
	C18:1 n-9 Oleic Acid	2290 ± 1147	42	2241 ± 1441	100	2095 ± 846	104	0.55	1.00	1908 ± 529	37	2026 ± 631	64	2032 ± 1577	96	0.85	1.00
	C20:1 n-9 Eicosenoic Acid	14.9 ± 8.7	42	14.0 ± 7.3	100	13.6 ± 6.4	105	0.62	1.00	13.4 ± 5.4	37	14.1 ± 5.9	64	13.5 ± 6.2	95	0.81	1.00
	C22:1 n-9 Docosenoic Acid	5.3 ± 4.4	35	4.2 ± 3.1	88	4.5 ± 4.1	100	0.33	1.00	4.5 ± 2.4	30	4.5 ± 3.5	57	4.2 ± 3.0	85	0.78	1.00
	C24:1 n-9 Nervonic Acid	81.1 ± 22.4	39	75.5 ± 20.6	98	72.9 ± 17.8	103	0.086	1.00	77.5 ± 15.6	37	78.5 ± 19.3	59	75.6 ± 16.9	92	0.58	1.00
PUFA	*C18:2 n-6 Linoleic Acid (LA)*	3725 ± 1127	42	3308 ± 806	100	3516 ± 845	105	*0.029*	0.76	3397 ± 814	37	3679 ± 859	65	3518 ± 887	96	0.26	1.00
	C18:3 n-6 gamma-Linolenic Acid (GLA)	57.9 ± 29.6	42	52.6 ± 25.1	99	53.3 ± 21.4	103	0.47	1.00	38.3 ± 15.9	37	44.1 ± 19.1	65	41.4 ± 20.4	96	0.33	1.00
	C20:2 n-6 Eicosadienoic Acid	22.5 ± 8.0	42	20.8 ± 7.9	100	21.4 ± 9.1	105	0.56	1.00	22.7 ± 9.2	37	24.2 ± 10.5	64	23.8 ± 14.0	95	0.84	1.00
	C20:3 n-6 Dihomo-gamma-Linolenic Acid (DGLA)	158 ± 50	42	153 ± 68.4	100	153 ± 57.4	105	0.92	1.00	159 ± 53.4	37	166 ± 57.7	65	153 ± 77.9	96	0.48	1.00
	***C20:4 n-6 Arachidonic Acid (AA)***	891 ± 255	42	790 ± 190	100	770 ± 203	105	***0.006***	**0.17**	780 ± 188	37	805 ± 219	65	735 ± 182	96	0.079	1.00
	***C22:4 n-6 Docosatetraenoic Acid (DTA)***	31.6 ± 10	42	27.3 ± 9.1	100	26.2 ± 8.5	105	***0.005***	**0.15**	24.5 ± 7.3	37	25.9 ± 8.7	65	23.8 ± 7.4	96	0.23	1.00
	*C22:5 n-6 Docosapentaenoic-6 Acid (DPA n-6)*	23.9 ± 9	42	20.6 ± 8.6	100	19.9 ± 8	105	*0.037*	0.89	24.1 ± 9.9	37	22.9 ± 9.6	65	21.1 ± 8.6	96	0.2	1.00
	C18:3 n-3 alpha-Linolenic Acid (ALA)	73.1 ± 53.2	42	66.8 ± 39.0	100	70.0 ± 49.1	105	0.75	1.00	60.3 ± 29.6	37	69.6 ± 34.9	65	67.4 ± 40.3	96	0.46	1.00
	C20:5 n-3 Eicosapentaenoic Acid (EPA)	46.1 ± 23.3	42	47.8 ± 34.7	100	45.5 ± 27.5	104	0.85	1.00	35.8 ± 23.7	37	42.0 ± 32.3	65	39.1 ± 24.2	96	0.54	1.00
	C22:5 n-3 Docosapentaenoic-3 Acid (DPA n-3)	47.5 ± 16.1	42	43.9 ± 15.0	100	43.1 ± 12.9	105	0.25	1.00	34.5 ± 9.5	37	40.4 ± 12.6	65	38.0 ± 11.8	96	0.054	1.00
	C22:6 n-3 Docosahexaenoic Acid (DHA)	130 ± 67.6	42	125 ± 59.8	100	120 ± 52.8	105	0.66	1.00	139 ± 53.9	37	151 ± 83.1	65	132 ± 57.9	96	0.23	1.00
	Σ24FFA	29343 ± 9546	42	26962 ± 8491	100	26932 ± 7471	105	0.23	1.00	26050 ± 6061	37	27611 ± 6426	65	26318 ± 7688	96	0.43	1.00
	Σ6SFA	4054 ± 1395	42	3741 ± 1409	100	3634 ± 12412	105	0.23	1.00	35734 ± 1010	37	3725.7 ± 996.2	65	3463 ± 1067	96	0.29	1.00
	Σ7MUFA	2793 ± 1322	42	2743 ± 1697	100	25597 ± 1125	105	0.54	1.00	2364 ± 655	37	2460 ± 820	65	2492 ± 1779	96	0.89	1.00
	*Σ11PUFA*	5206 ± 1461	42	4654 ± 1041	100	4838 ± 1091	105	*0.033*	0.83	4715 ± 1015	37	5070 ± 1087	65	4793 ± 1106	96	0.18	1.00
	Σw3PUFA ¶	296 ± 138	42	283 ± 122	100	279 ± 115	105	0.72	1.00	269 ± 91.2	37	303 ± 139	65	277 ± 106	96	0.27	1.00
	Σw6PUFA §	4910 ± 1347	42	4371 ± 968	100	4560 ± 1014	105	*0.023*	0.64	4445 ± 965	37	4767 ± 1012	65	4516 ± 1040	96	0.2	1.00
	w6/w3¶	17.8 ± 4.9	42	17.1 ± 4.8	100	17.9 ± 5.0	105	0.5	1.00	17.5 ± 4.4	37	17.1 ± 4.5	65	17.6 ± 4.8	96	0.79	1.00
	C16:1/C16:0	14.1 ± 4.6	42	14.0 ± 5.3	100	14.6 ± 6.0	105	0.72	1.00	13.6 ± 3.7	37	13.6 ± 4.2	65	14.0 ± 4.9	95	0.81	1.00

All values are in μmol/L. Significant values are shown in bold and italic fonts. Abbreviations: SFA: Saturated Fatty Acid; MUFA: Mono-Unsaturated Fatty Acid; PUFA: Poly-Unsaturated Fatty Acid. ¶ Σw3PUFA: Σ (SSALN_N, SSEPA_N, SSDP3_N, SSDHA_N ). ¶ Σw3PUFA: Σ (SSALN_N, SSEPA_N, SSDP3_N, SSDHA_N ) § Σw6PUFA: Σ (SSLNA_N, SSGLA_N, SSED1_N, SSHGL_N, SSARA_N, SSDTA_N, SSDP6_N).

**Table 4:** ANOVA and pairwise t-test p values comparing Low vs. Moderate + High CVFLs in men.

	ANOVA *p*	*t*-test *p*			
	L vs. M vs. H	L vs. M	L vs. H	M vs. H	L vs. (M + H)
Arachidic acid	<0.001	0.01	0.01	0.998	0.01
Docosanoic acid	<0.001	0.001	0.004	0.545	0.002
Arachidonic Acid	0.006	0.024	0.008	0.481	0.01
Docosatetraenoic Acid	0.005	0.021	0.003	0.346	0.037

Abbreviations: ANOVA (Analysis of Variance); L: low; M: moderate; H: high.

**Table 5:** Univariate and multivariate linear regression analysis in Men and Women separately.

Men																
*SSAR1_N*	C20:0 Arachidic Acid															
	SSAR1_N (log)		Age (yr)		Smoking (Y/N)		dBP		BMI		CRP		HOMA_IR		TG	
	β	*p*	β	*p*	β	*p*	β	*p*	β	*p*	β	*p*	β	*p*	β	*p*
**model0**	−0.235	**6e-7**	---	---	---	---	---	---	---	---	---	---	---	---	---	---
**model1**	−0.161	**0.002**	−0.004	**0.010**	0.002	0.94	−0.003	**0.025**	−0.001	0.64	---	---	---	---	---	---
**model2**	−0.161	**0.002**	−0.004	**0.011**	0.002	0.95	−0.003	**0.025**	−0.001	0.64	−0.001	0.94	---	---	---	---
**model3**	−0.154	**0.003**	−0.004	**0.007**	0.002	0.94	−0.003	**0.038**	−0.0004	0.92	−0.001	0.91	−0.007	0.36	---	---
**model4**	−0.167	**0.004**	−0.004	**0.009**	0.0004	0.99	−0.003	**0.036**	−0.001	0.84	−0.001	0.92	−0.008	0.33	6.6e-5	0.60
***SSDA1_N***	**C22:0 Docosanoic Acid**															
	SSDA1_N (log)		Age (yr)		Smoking (Y/N)		dBP		BMI		CRP		HOMA_IR		TG	
	β	*p*	β	*p*	β	*p*	β	*p*	β	*p*	β	*p*	β	*p*	β	*p*
**model0**	−0.182	**6e-5**	---	---	---	---	---	---	---	---	---	---	---	---	---	---
**model1**	−0.125	**0.012**	−0.004	**0.008**	−0.002	0.94	−0.003	**0.016**	−0.002	0.48	---	---	---	---	---	---
**model2**	−0.126	**0.012**	−0.004	**0.008**	−0.002	0.93	−0.003	**0.017**	−0.002	0.49	−0.003	0.77	---	---	---	---
**model3**	−0.123	**0.014**	−0.004	**0.004**	−0.002	0.95	−0.003	**0.029**	−0.001	0.84	−0.003	0.74	−0.009	0.23	---	---
**model4**	−0.121	**0.017**	−0.004	**0.007**	−0.001	0.98	−0.003	**0.030**	−0.0004	0.91	−0.003	0.74	−0.008	0.33	−5.5e-5	0.64
***SSARA_N***	**C20:4 *n*-6 Arachidonic Acid (AA)**															
	SSARA_N (log)		Age (yr)		Smoking (Y/N)		dBP		BMI		CRP		HOMA_IR		TG	
	β	*p*	β	*p*	β	*p*	β	*p*	β	*p*	β	*p*	β	*p*	β	*p*
**model0**	−0.178	**4e-5**	---	---	---	---	---	---	---	---	---	---	---	---	---	---
**model1**	−0.119	**0.018**	−0.004	**0.008**	0.012	0.65	−0.003	**0.039**	−0.002	0.53	---	---	---	---	---	---
**model2**	−0.119	**0.018**	−0.004	**0.008**	0.012	0.65	−0.003	**0.039**	−0.002	0.53	0.0002	0.98	---	---	---	---
**model3**	−0.115	**0.022**	−0.004	**0.005**	0.012	0.64	−0.002	0.064	−0.0004	0.90	−0.0002	0.98	−0.01	0.21	---	---
**model4**	−0.117	**0.026**	−0.004	**0.008**	0.012	0.65	−0.002	0.062	−0.0004	0.91	−0.0002	0.98	−0.009	0.28	−1.9e-5	0.88
***SSDTA_N***	**C22:4 *n*-6 Docosatetraenoic Acid (DTA)**															
	SSDTA_N (log)		Age (yr)		Smoking (Y/N)		dBP		BMI		CRP		HOMA_IR		TG	
	β	*p*	β	*p*	β	*p*	β	*p*	β	*p*	β	*p*	β	*p*	β	*p*
**model0**	−0.123	**3e-4**	---	---	---	---	---	---	---	---	---	---	---	---	---	---
**model1**	−0.084	**0.021**	−0.004	**0.004**	0.011	0.66	−0.003	**0.027**	−0.002	0.50	---	---	---	---	---	---
**model2**	−0.084	**0.021**	−0.004	**0.004**	0.011	0.67	−0.003	**0.027**	−0.002	0.50	−0.001	0.93	---	---	---	---
**model3**	−0.077	**0.039**	−0.005	**0.003**	0.010	0.68	−0.002	**0.041**	−0.001	0.76	−0.001	0.91	−0.007	0.35	---	---
**model4**	−0.088	**0.045**	−0.005	**0.004**	0.010	0.68	−0.003	**0.039**	−0.001	0.70	−0.001	0.91	−0.008	0.34	5.6e-5	0.68
**Women**																
***SSAR1_N***	**C20:0 Arachidic Acid**															
	SSAR1_N (log)		Age (yr)		Smoking (Y/N)		dBP		BMI		CRP		HOMA_IR		TG	
	β	*p*	β	*p*	β	*p*	β	*p*	β	*p*	β	*p*	β	*p*	β	*p*
**model0**	−0.098	0.21	---	---	---	---	---	---	---	---	---	---	---	---	---	---
**model1**	−0.097	0.30	−0.002	0.37	0.025	0.59	−0.003	0.18	−0.008	0.051	---	---	---	---	---	---
**model2**	−0.092	0.33	−0.002	0.35	0.026	0.57	−0.003	0.21	−0.008	0.08	−0.019	0.61	---	---	---	---
**model3**	−0.090	0.36	−0.002	0.33	0.027	0.57	−0.003	0.22	−0.007	0.13	−0.013	0.73	−0.007	0.64	---	---
**model4**	−0.105	0.33	−0.002	0.37	0.026	0.58	−0.003	0.21	−0.008	0.12	−0.016	0.69	−0.009	0.59	1e-4	0.74
***SSDA1_N***	**C22:0 Docosanoic Acid**															
	SSDA1_N (log)		Age (yr)		Smoking (Y/N)		dBP		BMI		CRP		HOMA_IR		TG	
	β	*p*	β	*p*	β	*p*	β	*p*	β	*p*	β	*p*	β	*p*	β	*p*
**model0**	−0.165	**0.022**	---	---	---	---	---	---	---	---	---	---	---	---	---	---
**model1**	−0.113	0.17	−0.002	0.43	0.030	0.52	−0.003	0.18	−0.009	**0.029**	---	---	---	---	---	---
**model2**	−0.122	0.14	−0.002	0.42	0.031	0.51	−0.003	0.23	−0.008	0.06	−0.030	0.42	---	---	---	---
**model3**	−0.121	0.15	−0.002	0.40	0.031	0.50	−0.003	0.24	−0.008	0.10	−0.024	0.54	−0.007	0.65	---	---
model4	−0.126	0.15	−0.002	0.42	0.032	0.51	−0.003	0.23	−0.008	0.10	−0.027	0.51	−0.008	0.62	1e-4	0.80
***SSARA_N***	**C20:4 *n*-6 Arachidonic Acid (AA)**															
	SSARA_N (log)		Age (yr)		Smoking (Y/N)		dBP		BMI		CRP		HOMA_IR		TG	
	β	*p*	β	*p*	β	*p*	β	*p*	β	*p*	β	*p*	β	*p*	β	*p*
**model0**	−0.147	**0.017**	---	---	---	---	---	---	---	---	---	---	---	---	---	---
**model1**	−0.15	**0.038**	−0.002	0.32	0.043	0.33	−0.003	0.21	−0.007	0.10	---	---	---	---	---	---
**model2**	−0.147	**0.042**	−0.002	0.30	0.044	0.32	−0.003	0.25	−0.006	0.15	−0.018	0.61	---	---	---	---
**model3**	−0.148	**0.045**	−0.002	0.30	0.044	0.33	−0.003	0.25	−0.006	0.20	−0.016	0.67	−0.003	0.84	---	---
**model4**	−0.163	**0.040**	−0.002	0.34	0.045	0.31	−0.003	0.21	−0.006	0.17	−0.021	0.59	−0.005	0.75	2e-4	0.59
***SSDTA_N***	**C22:4 *n*-6 Docosatetraenoic Acid (DTA)**															
	SSDTA_N (log)		Age (yr)		Smoking (Y/N)		dBP		BMI		CRP		HOMA_IR		TG	
	β	*p*	β	*p*	β	*p*	β	*p*	β	*p*	β	*p*	β	*p*	β	*p*
**model0**	−0.043	0.40	---	---	---	---	---	---	---	---	---	---	---	---	---	---
**model1**	−0.093	0.11	−0.003	0.19	0.046	0.31	−0.003	0.23	−0.007	0.11	---	---	---	---	---	---
**model2**	−0.090	0.13	−0.003	0.18	0.046	0.31	−0.003	0.25	−0.006	0.14	−0.013	0.72	---	---	---	---
**model3**	−0.089	0.14	−0.003	0.18	0.046	0.31	−0.003	0.26	−0.006	0.18	−0.011	0.77	−0.003	0.84	---	---
**model4**	−0.129	0.09	−0.003	0.23	0.052	0.26	−0.003	0.18	−0.007	0.13	−0.017	0.66	−0.006	0.69	4e-4	0.37

The effects of various combinations of potential confounding factors were adjusted with multivariate models as follows: Model 0: log VO2max ~ log FA (univariate analysis); Model 1: log VO2max ~ log FA + Age + Smoking + dBP + BMI; Model 2: log VO2max ~ log FA + Age + Smoking + dBP + BMI + CRP; Model 3: log VO2max ~ log FA + Age + Smoking + dBP + BMI + CRP + HOMA-IR; Model 4: log VO2max ~ log FA + Age + Smoking + dBP + BMI + CRP + HOMA-IR + TG; Abbreviations: dBP: diastolic Blood Pressure (mmHg); BMI: Body Mass Index (kg/m^2^); CRP: Plasma C-Reactive Protein (mg/dL); β: coefficient of variable; HOMA_IR: HOmeostatic Model Assessment – Insulin Resistance; TG: Plasma Triglyceride (mg/dL).
